# Role of a metastatic suppressor gene KAI1/CD82 in the diagnosis and prognosis of breast cancer

**DOI:** 10.1016/j.sjbs.2021.03.001

**Published:** 2021-03-10

**Authors:** Khulood M. Al-Khater, Sarah Almofty, Vijaya Ravinayagam, Noor Alrushaid, Suriya Rehman

**Affiliations:** a Department of Anatomy, College of Medicine, Imam Abdulrahman Bin Faisal University, P.O. Box 1982, Dammam 31441, Saudi Arabia; b Department of Stem Cell Biology, Institute of Research and Medical Consultations (IRMC), Imam Abdulrahman Bin Faisal University, Dammam 31441, Saudi Arabia; c Deanship of Scientific Research, and Department of Nanomedicine, Institute of Research and Medical Consultations (IRMC), Imam Abdulrahman Bin Faisal University, Dammam 31441, Saudi Arabia; d Department of Epidemic Disease Research, Institute of Research and Medical Consultations (IRMC), Imam Abdulrahman Bin Faisal University, Dammam 31441, Saudi Arabia

**Keywords:** Tumor, Breast cancer, Metastasis, KAI1/CD82, Metastasis suppressors, Protein

## Abstract

Globally, breast cancer is the most common type of cancer in females and is one of the leading causes of cancer death in women. The advancement in the targeted therapies and the slight understanding of the molecular cascades of the disease have led to small improvement in the rate of survival of breast cancer patients. However, metastasis and resistance to the current drugs still remain as challenges in the management of breast cancer patients. Metastasis, potentially, leads to failure of the available treatment, and thereby, makes the research on metastatic suppressors a high priority. Tumor metastasis suppressors are several genes and their protein products that have the capability of arresting the metastatic process without affecting the tumor formation. The metastasis suppressors KAI1 (also known as CD82) has been found to inhibit tumor metastasis in various types of solid cancers, including breast cancer. KAI1 was identified as a metastasis suppressor that inhibits the process of metastasis by regulating several mechanisms, including cell motility and invasion, induction of cell senescence, cell–cell adhesion and apoptosis. KAI1 is a member of tetraspanin membrane protein family. It interacts with other tetraspanins, chemokines and integrins to control diverse signaling pathways, which are crucial for protein trafficking and intracellular communication. It follows that better understanding of the molecular events of such genes is needed to develop prognostic biomarkers, and to identify specific therapies for breast cancer patients. This review aims to discuss the role of KAI1/CD82 as a prognosticator in breast cancer.

## 1 Introduction

Carcinogenesis is a complex pathological process that involves conversion of normal cells into a highly dividing ones that can spread beyond the normal limit of the original site. Cancer incidence is increasing all over the world, and it has been reported that more than 19 million new cancer cases were diagnosed in 2020, 11.7% of which were breast cancer cases (Cancer Today, n.d). Metastasis of cancer cells (i.e., spread of cells beyond the limits of the original site) is considered one of the main challenges in management of cancer patients. Most of cancer deaths occur due to metastasis to secondary sites (Just, 2011, Cancer, n.d.). The process of progression of the cancer from the status of locally-confined growth to the stage of dissemination into distant areas is known as the metastasis cascade (Tsai and Weissman, 2011). This cascade involves several stages starting from invasion of blood or lymph vessels and ending at the colonization of the distant site. The underlying molecular mechanism of metastasis is attracting the attention of researchers during the last few decades. The purpose is to find a therapeutic strategy that could prevent metastasis of cancer cells, and hence, improve the prognosis of the disease. A very interesting breakthrough in cancer research is the discovery of genes capable of suppressing or promoting the metastasis of cancer cells (Yoshida et al., 2000, Stafford et al., 2008). Researchers called the former genes, and their protein products, metastasis suppressors, and the definition given to these genes is “a group of genes capable of inhibiting the metastasis but having no effect on the development of cells in the primary site” (Just, 2011). Many of these were identified and examples are CD44, Kangai1 protein (KAI1), MKK4, Nm23-H1 and breast cancer metastasis suppressor 1 (BRMS1) (Just, 2011), and KiSS-1 (Lee et al., 1996). This review focuses on one of these metastasis suppressors, the KAI1 (also known as CD82), and an up-to-date account on its effect on the diagnosis and prognosis of breast cancer patients is presented. The literatures were collected from Scopus, Web of Science and PubMed.

## 2 KAI1 structure

KAI1 (CD82) was originally identified as a surface protein on lymphocytes involved in activation of T cells (Lebel-Binay et al., 1995). Its first recognition as a metastasis suppressor was in 1995 after the work accomplished by Dong and co-workers who reported transfection of this gene into rat prostate cancer cells led to suppression of metastasis (Dong et al., 1995). Furthermore, these researchers demonstrated that the KAI1 gene was down-regulated in cells obtained from human metastatic prostate cancer (Dong et al., 1995, Dong et al., 1996). In an attempt to describe the mechanism of action of this gene, Dong et al. (1997) presented a characterization of its various regions, which consist of 10 exons and 9 introns; the coding region starts from exon 3 to exon 10. Other research group from Japan agreed with this observation after examining the expression of KAI1 gene in human prostate cancer (Ueda et al., 1996). In addition to KAI1/CD82 role of mediating tumor aggression, the protein was observed to play a critical role in virus receptor coordination (binding) and entry into cells. The expression of KAI1/CD82 tends to be altered in different cancers and has been linked to survival term of patients. A decrease in CD82 level was found to induce aggressive tumor progression, while an increased level suppressed the tumor spread (secondary tumor). The cell invasion and migration phenomenon were shown to subdue with high level of KAI1/CD82 leading to a decrease in cellular process of engulfing (endocytosis) of epidermal growth factor receptors (Malik et al., 2013). Reduced expression of KAI1 was also described in cases of metastasis of other solid tumors like cancer of lung (Adachi et al., 1996, Takeda et al., 2007), bladder (Yu et al., 1997, Jackson et al., 2000), pancreas (Guo et al., 1996, Xu et al., 2008), liver (Yang et al., 2008), cervix (Liu et al., 2001), kidney (Zhu et al., 2017b), and breast (Yang et al., 2001), as well as in sarcoma (Tsai et al., 2007). Tonoli and Barrett (2005) presented a review of various tumor types in which downregulation of KAI1 was associated with metastasis of cancer cells.

**Fig. 1:**
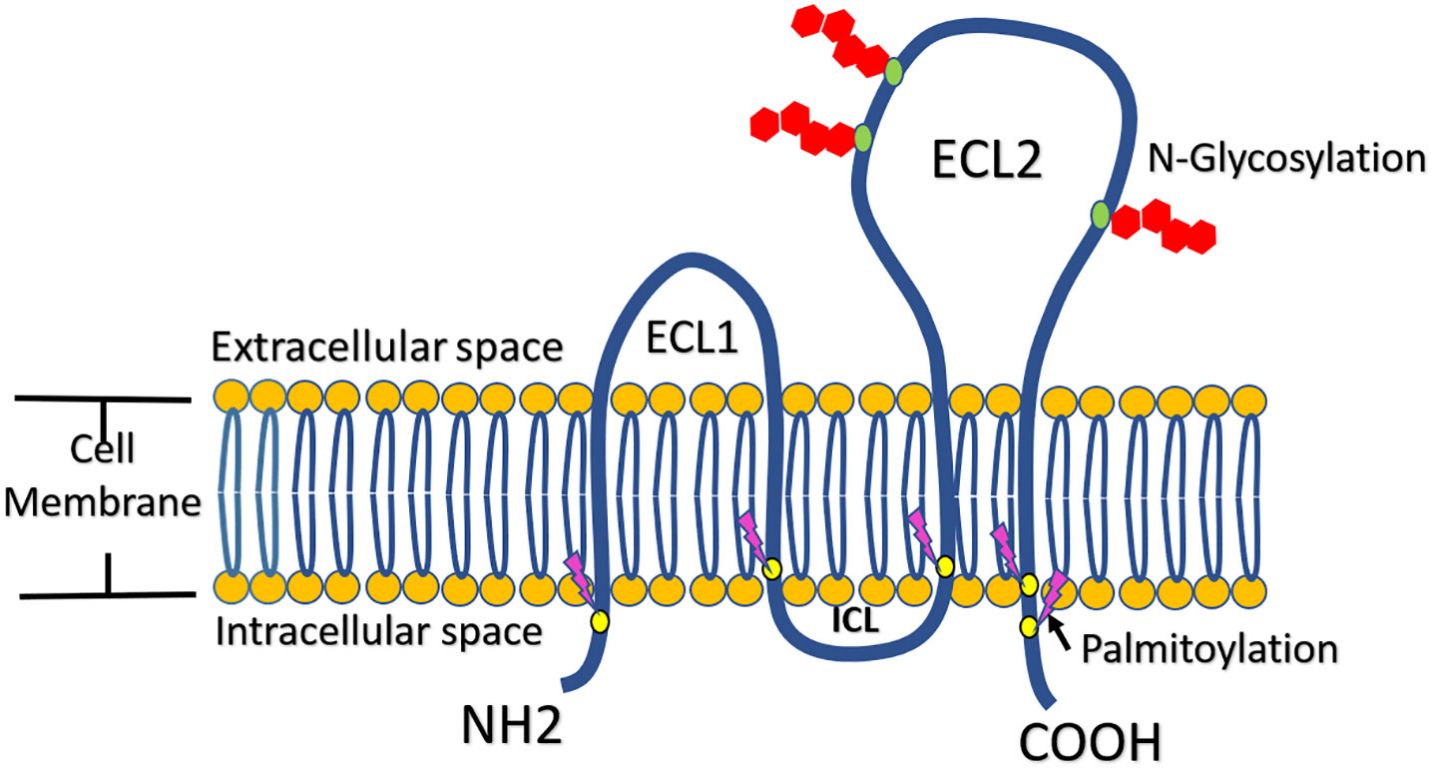
This schematic drawing represents the structure of KAI1 protein, a transmembrane protein with 2 intracellular terminals: amine- (NH2) and carboxyl- (COOH) ends, a small intracellular loop (ICL), a small extracellular loop (ECL1), a large extracellular loop (ECL2) and 4 transmembrane domains. Post-translational modifications, N-glycosylation (at 3 points on ECL2) and palmitoylation (at 5 points near the cytosol) are shown.

The KAI1 gene encodes a protein, also known as KAI1, which is a membrane glycoprotein that belongs to the transmembrane 4 superfamily (TM4SF) or tetraspanins. There are 33 different tetraspanins identified in the genome of eukaryotic cells (Detchokul et al., 2014). KAI1 protein is formed of 267 amino acids arranged as 2 intracellular terminals (N- and C-terminal domains), a short intracellular loop of 4 amino acids and two extracellular loops, one short (ECL1) and one long (ECL2) (Fig. 1). The cysteine residues in the ECL2 are believed to form three disulfide bonds, and the ECL2 loop also contains three N-linked glycosylation spots. KAI1, like other tetraspanins, forms huge multimeric complexes with other tetraspanins, in addition to cytosolic and membrane proteins like integrins, tyrosine kinases and adaptor proteins that play part in many cascades of signaling (Tsai and Weissman, 2011). It is presumed that KAI1 has no enzymatic activity (Tsai and Weissman, 2011).

## 3 KAI1 function

A variety of cellular processes are regulated by KAI1, and potentially relevant to metastasis suppression are cell–cell adhesion, cell motility and invasion, cell aging, cell death and survival (Tsai and Weissman, 2011).

### 3.1 Cell-cell adhesion

A prominent role of KAI1 is to encourage homotypic cell–cell adhesion and loss of this function plays an important part in metastasis and epithelial–to-mesenchymal transition. In DU 145 human prostate cancer cells, for example, transfection of KAI1 led to homotypic cell–cell aggregation (Jee et al., 2003). Another example is demonstrated in non-small cell lung cancer, where KAI1 augmented intercellular adhesion of cancer cells (Abe et al., 2008). This effect was attributed to the influence on E-cadherin/β-catenin complex, which is stabilized by KAI1 expression (Abe et al., 2008). As a result, metastasis was inhibited and cancer cells were confined to the primary site. In human bladder cancer cell lines, Jackson and co-workers demonstrated that low expression of KAI1 was associated with reduced adhesion to fibronectin, but not to laminin or collagen type IV, in the extracellular matrix (ECM) (Jackson et al., 2000).

### 3.2 Cell motility and invasion

Several *in vitro* studies demonstrated that overexpression of KAI1 was associated with inhibition of cellular motility and invasiveness. Integrins, receptors for laminin and fibronectin, are known to mediate cell adhesion to the ECM. It has been shown that expression of KAI1 in Du 145 prostate cancer cells led to down-regulation of α6 integrin resulting in decreased cellular migration and abolished cell morphogenesis (He et al., 2005). This effect occurred due to internalization of the integrin, a feature that explains the down-regulation of this receptor in these cells (He et al., 2005). Another study suggested that the effect of KAI1 is mediated by attenuating the epidermal growth factor receptor (EGFR), which is important for cell migration through stimulation of the internalization of the activated receptor (Odintsova et al., 2000). An important role of KAI1 in inhibiting cell motility is by regulating the activity of receptor tyrosine kinases (RTKs) (Tsai and Weissman, 2011). One of these RTKs, known as c-Met, as well as the proto-oncogene cytoplasmic tyrosine kinase Src, and its substrates p130Cas were found to be down-regulated in prostate cancer cells that express KAI1 (Sridhar and Miranti, 2006). The research work conducted by Lee and co-workers suggested that the C-terminal end of KAI1 is the main part that regulates the function of this protein (Lee et al., 2003). These authors identified a protein, known as KITENIN, that interacts with the C-end of KAI1 in order to make a balance between invasiveness and anti-invasiveness of the cells in a murine model of colon cancer cell line (Lee et al., 2004). KITENIN stands for KAI1 COOH-Terminal Interacting Tetraspanin, and its expression is believed to decrease the metastasis suppression role of KAI1, and consequently increase the invasiveness of cancer cells (Lee et al., 2004).

Cancer associated with AIDS is known as Kaposi’s sarcoma (KS). The cancer caused by KV associated herpesvirus (KSHV) has been reported with capability of forming new blood vessels and lymphatic invasive tumor (Li et al., 2020a, Li et al., 2020b). Viral interferon regulatory factor 1 (vIRF1) is a protein composed on 449 amino acids is reported to involve in cell migration (cell motility). The active role of such protein has been shown in angiogenesis by upregulating transmembrane glycoprotein; complement C1r/C1s, Uegf, Bmp1 (CUB containing protein 1). Lymphoid enhancer-binding factor 1 derived as (Lef1) is reported to be present in pre-B and T cells. vIRF1 reported to express such 48-kD nuclear protein (Lef1) and involve in binding and promoting CDCP1 transcription. vIRF1 protein is also known to suppress CD82, which is known to be a metastasis suppressor via ubiquitin–proteasome pathway. The vIRF1 use E3 biquitin ligase AMFR to suppressor CD82 and thereby protects CDCP1 from degradation through palmitoylation mediated by CD82. Therefore, the CUB domain containing protein activates the AKT signaling for cell mobility rather than angiogenesis. The critical role of vIRF1 in mitigating the cellular protein and pathways has been identified that suppress the role of Lef1 and CD82 and activates CDCP1 towards angiogenesis and cell migration (W. Li et al., 2020).

Using human ovarian cancer cell lines (ES2 and SKOV3) and clinical samples from ovarian cancer patients, (J. Li et al., 2020) demonstrated that glycosylation of KAI1 at a specific residue (Asn 157) is a crucial post-translational modification essential for inhibition of ovarian cancer metastasis by KAI1. The glycosylation of KAI1 led to disruption of the integrin-fibronectin interaction, a signaling pathway important for cell migration. This was also confirmed using *in vivo* technique applied on BALB/c nude mice subcutaneously injected with ES2 cells (ovarian cancer cells) (J. Li et al., 2020). This last study also reported that the enzyme glycosyltransferase MGAT3 catalyzed the glycosylation of KAI1.

In addition, they documented that treating ovarian cells with KAI1-enriched exosomes efficiently prevented *in vitro* adhesion and migration of ovarian cancer cells, and this may signify a new strategy for treating ovarian cancer metastasis (J. Li et al., 2020).

### 3.3 Cell aging

The Duffy antigen/receptor for chemokines (DARC), which is an endothelial cell surface protein, has been recognized as an interacting partner of KAI1 (Bandyopadhyay et al., 2006). Tumor cells expressing KAI1 showed enhanced adhesion to endothelial cells, and it has been proposed that the interaction between KAI1 and DARC induces aging in tumor cells, and this reduces the metastasis potential (Bandyopadhyay et al., 2006). DARC has also been described to have a role in causing cellular aging by attenuating the angiogenesis because of its ability to remove the angiogenic factors secreted by tumor cells (Shen et al., 2006, Xu et al., 2007). Shen et al. (2006) found that the growth of prostate cancer in DARC-deficient mice was more aggressive, compared to wild-type mice, due to the unblocked angiogenesis of tumor cells. However, this link between DARC and malignancy has been debated by one study (Elson et al., 2011).

### 3.4 Cell death and survival

It is well known that an important factor in metastasis formation is the ability of tumor cells to survive in the circulation and at the secondary colonization sites. As the tumor cells migrate from the primary tumor site, the loss of adhesion to the ECM may lead to apoptosis in the circulating tumor cells (Tsai et al., 2007). This was observed in tumor cells in the lung following venous injection of fibrosarcoma cells into mice deficient in the protease ligase gp78, and this was explained by the increased effect of KAI1 (Tsai et al., 2007). These authors reported that gp78 plays a role in post-translational degradation of KAI1, hence, absence of gp78 leads to augmentation of function of KAI1 as a metastasis suppressor and promoting apoptosis in tumor cells. Other studies have also reported the role of KAI1 in induction of cell death (Ono et al., 1999, Schoenfeld et al., 2004, Zismanov et al., 2009). One of these studies showed that in Chinese hamster ovary mutant cell line ldlD-14 deficient in UDP-Glc 4-epimerase, high levels of KAI1 expression promoted cell death (apoptosis) after 11 days (Ono et al., 1999). Ono and colleagues attributed this observation to post-translational modification of CD82 by N-glycosylation and synthesis of an endogenous GM3 (ganglioside) (Ono et al., 1999). KAI1-induced apoptosis observed in multiple myeloma cell line was explained by autophagy and activation of unfolded protein response (UPR) signaling pathway secondary to endoplasmic reticulum stress (Zismanov et al., 2009). In 2004, the research group of Grimm reported that KAI1 induced apoptosis in tumor cells secondary to oxidative stress produced by generation of reactive oxygen intermediates (ROIs) and efflux of cellular glutathione (Schoenfeld et al., 2004).

## 4 Mechanism of downregulation of KAI1 in metastasis

**Fig. 2:**
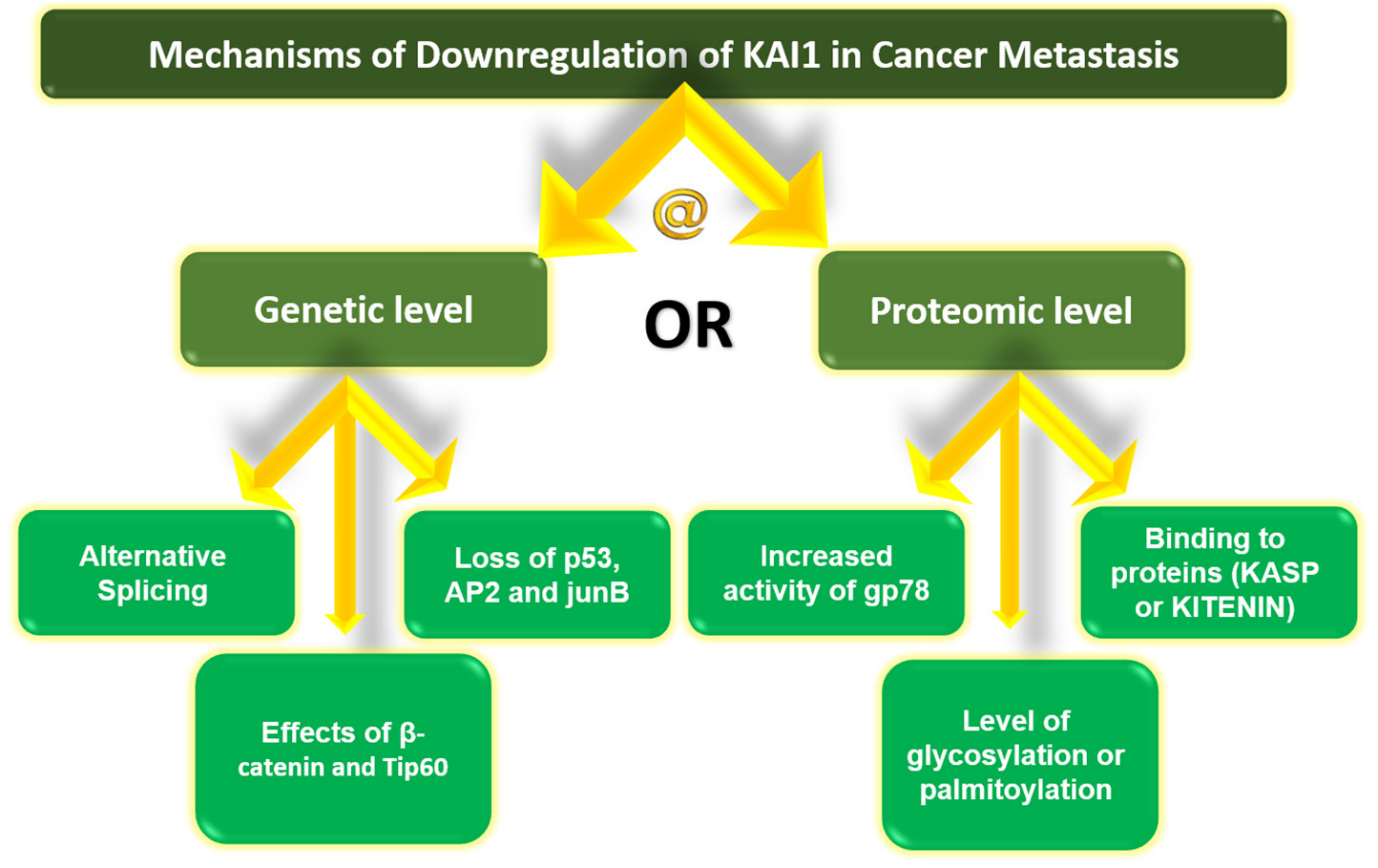
Summary of the main mechanisms of KAI1 downregulation in metastasis described in the literature.

Various mechanisms have been proposed for the downregulation of the KAI1 in cancer cells that occur in cases of metastasis (Fig. 2). These probably act at two different levels: genomic or proteomic (i.e., at the protein) levels (Tonoli and Barrett, 2005). One of the mechanisms that occur at the genomic level is the alternative splicing, which means alteration in the gene expression by addition or removal of one or more exon regions of the gene in the transcribed mRNA (Keren et al., 2010). This leads to change in the amino acid sequence of the resulting protein, a feature that would change its function. This alternative splicing was reported in cells of gastric cancer (Lee et al., 2003) and ovarian cancer (Upheber et al., 2015). Lee et al. (2003) described the presence of a spliced variant of KAI1 in advanced cases of gastric cancer patients with poor prognosis. The deleted part of the transcribed mRNA of KAI1 is exon 7 that encodes 28 amino acids in KAI1 protein. The resulting abnormal KAI1 was found to have weak interaction with proteins important for cell–cell adhesion like integrin. Furthermore, similar results were found in mouse colon adenocarcinoma with high invasiveness (Lee et al., 2003). Upheber et al. (2015) described the presence of a spliced variant of KAI1 in ovarian cancer cells, and this was associated with reduced integrin-mediated cell adhesion leading to migration of tumor cells. A spliced variant KAI1 has been also documented in various stages of human bladder cancer, as well as cancer cell lines; however, this variant was not associated with the degree of invasiveness of the cancer (Jackson et al., 2007). Loss of heterozygosity, i.e., loss of one of the alleles of KAI1 gene on chromosome 11p11.2, is another mechanism described in the literature (Kim et al., 2009), though evidence for this mechanism was not provided by a previous study (Tagawa et al., 1999). Other postulated mechanisms like gene mutation, promoter mutation, or hypermethylation of the CpG island of the promoter (part of the KAI1 gene) were not supported by enough evidence (Tonoli and Barrett, 2005). In prostate cancer cell lines, the KAI1 gene has been found to be activated by three transcription factors (TFs): p53, AP2, and junB; this implies that loss of function of these TFs would lead to downregulation of KAI1 and, consequently, metastasis of tumor cells (Marreiros et al., 2005). One of the proteins encoded by the gene p63, known as delta-N-p63-alpha, is found to have an influence on KAI1 by upregulating its expression (Wu et al., 2014). In hepatocellular carcinoma, the expression of KAI1 was found to be increased by antagonizing miRNA 197, one of the micro-RNAs that are involved in carcinogenesis (Dai et al., 2014). Downregulation of KAI1 in melanoma cell lines A375 and B16 was found to be associated with overexpression of another micro-RNA, known as miRNA 633 (Wang and Yaling, 2021). Therefore, authors postulated that micro-RNA 633 binds with KAI1 and impairs its function through regulation of its promoter activity (Wang and Yaling, 2021). A recent study was conducted on genetically engineered mice to elucidate the mechanism of melanoma metastasis (Marie et al., 2020). This study reported that some embryonic genes were activated and these were responsible for the invasion of melanoma cells. Specifically, KDELR3 gene has been reported to regulate the post-translational glycosylation and degradation of KAI1 (Marie et al., 2020). In prostate cancer cells, Kim et al. (2005) reported that a balance between the repressive effect of β-catenin and the activating effect of the Tip60, a histone acetyltransferase enzyme, on the promoter region of KAI1 is needed to control the expression of this gene and its metastatic potential. A functional hypoxia-response element has been identified in the promoter of KAI1 gene of mice, and this is modulated by hypoxia-inducible factor 1α (HIF-1α), this suggests that KAI1 is involved in hypoxia (Kim et al., 2010). Other investigations on prostate cancer cell lines showed that KAI1 prevents metastasis through maintaining the integrity of E-cadherin, and this was explained by suppressing the proteolytic activity of ADAM17, a member of disintegrin and metalloprotease (ADAM) family that enhances tumor spread (Ma et al., 2020). The other group pf postulated mechanisms of KAI1 downregulation occurs at the proteomic level, for example, post-translational modification of proteins important for adhesion of cells to the ECM, like integrin and fibronectin, as demonstrated on human prostate cell lines (Lee et al., 2011). Post-translational modification of KAI1 protein has been reported secondary to increased activity of gp78, an ubiquitin ligase located in the membrane of the endoplasmic reticulum that causes degradation of KAI1, and therefore, reduction in its expression (Tsai et al., 2007). The level of glycosylation or palmitoylation of KAI1 has also been suggested to affect its level of expression in cancer cells (Tonoli and Barrett, 2005). Direct binding to certain membrane proteins, like KASP or KITENIN was reported to have an influence on the activity of KAI1 (Tonoli and Barrett, 2005). KAI1 has been implicated in the mechanism of brain metastasis from lung cancer. Using human pulmonary adenocarcinoma brain metastasis cell line PC-14/B, Jiang et al. (2020) reported that suppression of KAI1 was associated with SIRT1-mediated metastasis. SIRT1 (also known as sirtuin 1) is a target gene for hsa-miR-217, one type of microRNAs (miRNAs) that play an important role in the metastasis of cancers. The investigators postulated that the axis of hsa-miR-217/sirtuin 1/P53/KAI1 pathway regulates the metastasis of non-small cell lung cancer to the brain. In a recent *in vitro* study using breast cancer and head and neck squamous cell carcinoma cell lines, a unique relation has been demonstrated between liprin-α1, a member of protein tyrosine phosphatase, and KAI1 (Pehkonen et al., 2018). This study reported that Liprin-α1 decreased KAI1 expression, and this enhanced the invasiveness of cancer cells. Zhu et al. (2017a) reported that KAI1 produced its inhibitory role in renal cell carcinoma cell lines (caki-1, caki-2, and 786-O) partially by decreasing the expression of TGF-β1/Smad pathway. TGF-β1 is a transforming growth factor-β that plays an important role in inducing the extracellular proteases MMPs (metalloproteinases). TGF-β1 activates transcription factors Smad 2/3 that regulate the expression of TGF-β target gene. Similar results were published by a study conducted on human prostate cancer cells. This latter study showed that KAI1 produced it effects by inhibiting the signals that promote the epithelial-to-mesenchymal transition of epithelial cells (Lee et al., 2019). The suppressed signals were the TGF‐β1/Smad and Wnt/β‐catenin signal pathways. A role for the gangliosides in promoting the anti-metastasis effect of KAI1 was postulated by Li et al. (2013). This suggestion was confirmed by a recent study that found the two gangliosides, GM2 and GM3, augmented the inhibitory effect of KAI1 on cell migration in human colorectal adenocarcinoma cell line SW620 (Huang et al., 2020). This effect was brought about by enhancing the inhibitory influence of KAI1 on the phosphorylation, and therefore, the activity of, EGFR by two different mechanisms: the inhibition of phosphorylation by GM3 occurred at both the Tyr1045 and Tyr1173 residues, but this happened only at Tyr1045 residue by GM2 (Huang et al., 2020). In addition, downregulation of MAPK signaling pathway was reported in both gangliosides. Via *in vitro* (Human ovarian cancer cell lines ES2 and SKOV3 and fresh samples from ovarian cancer patients) and *in vivo* (nude mice) approaches, J. Li et al. (2020) found that the proper functioning of KAI1 requires post-translational glycosylation at Asn157 site. This glycosylation is produced by the glycosyltransferase MGAT3. Downregulation of MGAT3 has been found in ovarian cancer with metastasis (J. Li et al., 2020).

The aforementioned account shows a variety of mechanisms that researchers described for the downregulation of KAI1 in cancer metastasis. It is possible that the mechanisms, and therefore, the targets for potential therapeutics, differ according to the type of cancer.

Despite the lack of full understanding of the underlying mechanisms of the function of KAI1 in inhibiting metastasis of cancer cells, attempts have been made in order to use KAI1 in therapeutic strategies to treat cancer patients (Tonoli and Barrett, 2005). These strategies might target the KAI1 at the genomic or proteomic levels. The earliest attempt was undertaken by Dong et al. (1995) after introducing the KAI1 gene into rat AT6.1 prostate cancer cells, and this was found to suppress the metastasis. Another attempt was performed in 2007 when a group of investigators transferred the KAI1 gene into lung tumor of mice (Takeda et al., 2007). They noticed dramatic reduction in the metastasis of cancer cells to mediastinal lymph nodes. Dai et al. (2014) succeeded in inhibiting the migration and invasion of hepatocellular carcinoma cells in *in vitro* and *in vivo* sittings after antagonizing the miR-197, a molecular manipulation that led to upregulation of KAI1.

Using a combination of patient samples and cell lines of acute myeloid leukemia (AML), Floren et al. (2020) demonstrated that KAI1 contributed to the chemo-resistant type of AML; KAI1 overexpression was associated with significant reduction in cell death following chemotherapy (daunorubicin). They described the mechanism of this resistance to chemotherapy as downstream activation of the β1 integrin and enhanced protein kinase c alpha (PKCα) signaling, and this led to activation of p38 mitogen-activated protein kinase (MAPK). Furthermore, KAI1 expression promoted the formation of dense β1 integrin membrane clusters. Therefore, these authors suggested that the KAI1-PKCα signaling axis may be a potential target for weakening chemo-resistance signaling in AML.

## 5 KAI1 as a metastasis suppressor in breast cancer

Worldwide, breast cancer is the most common type of cancer in the females, and it is the leading cause of death in cancer patients in many countries (Bray et al., 2018). Pathologically, there are four stages of breast cancer and this staging method depends on the TNM (Tumor-Node-Metastasis) system. The T describes the location and size of the primary tumor, N refers to the status of the regional lymph nodes, and M indicates whether distant metastasis is present (Cserni et al., 2018). It has been estimated that about 6% of breast cancer patients have already developed metastasis of cancer cells at the time of diagnosis (Breast Cancer - Metastatic: Statistics | Cancer.Net, n.d). Metastasis is generally associated with poor prognosis of the disease. As presented in the previous account of this review, number of genes/proteins have been implicated in the suppression of the metastatic activity of cancer cells. During the last few decades, researchers have been trying to unravel the complexity of this area of research by identifying these genes/proteins aiming to find strategies to prevent the process of metastasis and improve the prognosis of the disease. One of the earliest attempts was conducted in 1996, when researchers reported that the proposed metastasis suppressor gene for breast cancer is located on chromosome 11p11.2 (Phillips et al., 1996). Subsequent studies were able to identify the specific gene responsible for the suppression of metastasis in breast cancer patients, and this gene was found to be KAI1 (Yang et al., 1997, Yang et al., 2000). These studies described downregulation of KAI1 gene with progression of breast cancer and occurrence of metastasis. Yang et al. (1997) demonstrated this finding by examining the levels of KAI1 mRNA in different breast cancer cell lines representing various stages of the disease, in addition to normal breast cells. They found that the more advanced stages showed lower expression of KAI1 and vice versa (Yang et al., 1997). Using immunohistochemistry and Western blot, the levels of KAI1 protein in breast cancer cell lines were found to match those of KAI1 mRNA reported in the previous study (Yang et al., 2000). Moreover, similar results were documented after examining specimens from 81 breast cancer patients (Yang et al., 2000). Yang et al. (2001) reported that malignant breast cancer cell lines transfected with KAI1 gene showed reduced invasiveness and suppression of the metastasis. This inverse relation between expression of KAI1 and stage of breast cancer was also documented in Chinese (Han et al., 2015), Pakistani (Mooez et al., 2011), and Saudi (Kussaibi et al., 2019) patients, as well as in other studies performed in Japan (Huang et al., 1998) and India (Latha et al., 2019). Huang et al. (1998) reported a relation between the low KAI1 expression and recurrence of breast cancer. Similar results were also reported by a study performed on 83 Indian breast cancer females, when researchers found a significant reduction in the expression of KAI1 in the metastatic cases compared to the non-metastatic ones (Singh et al., 2016). They concluded that reduction in the expression of KAI1 is correlated with aggressiveness and, consequently, poor prognosis of breast cancer. As a result, these authors suggested that measuring the expression level of KAI1 can be used as a marker for the prognosis of breast cancer and a guidance to plan therapeutic measures for those patients. Latha et al. (2019) have studied the metastasis suppressor genes KAI-1/CD82. Though the role of KAI1 has been much understood with prostate cancer, it is not explored well in breast cancer. They studied CD82 expression at both transcriptional and translational levels in benign and breast cancer patients. A relationship has been explored between the KAI1 expression levels and clinicopathological parameters. A significant decrease in the expression of KAI1 [protein levels (P < 0.05) and gene] was observed with breast cancer than benign breast disease. Furthermore, the expression levels of KAI1 have been strongly related with the status of lymph node and advanced tumor stages (P < 0.05). No relation has been found with age, receptor status (ER, PR and Her2), age, and tumor grade. The study showed that the status of lymph node metastasis and tumor staging can be correlated with the lower expression of KAI1 and can predict the prognosis of breast cancer. In a recent *meta*-analysis of 29 eligible studies, Zhu and co-investigators found that KAI1 can be regarded as a promising biomarker to predict the prognosis of many types of cancer, including breast cancer (Zhu et al., 2017b), as they reported that the overall survival of cancer patients increased significantly with the positive expression of KAI1. However, these authors advised that their data should be interpreted with caution due to some unavoidable limitations in the *meta*-analysis related to certain issues like heterogeneity of the patients (e.g. age, tumor type, ethnicity) included in the studies. In a recent study, researchers recommended using non-invasive methods to diagnose and assess the progress of breast cancer; an example of these methods is using what they called “liquid biopsy” obtained from body fluids (e.g. blood, urine) (Wang et al., 2019). This recommendation is based on their finding that the KAI1 is expressed on exosomes that are excreted into body fluids, and this exosomal KAI1 had an inverse relation with the stage of breast cancer; the more advanced stages showed lower expression of exosomal KAI1.

In an attempt to investigate the mechanism underlying the downregulation of KAI1 in breast cancer, a process that precedes metastasis, a group of investigators from Saudi Arabia studied the expression of KAI1 in 90 formalin-fixed breast cancer tissue obtained from Saudi patients (Kussaibi et al., 2019). They used 2 antibodies against KAI1; one targeted the carboxyl terminal of the protein, whereas the other one was against the large extracellular loop. The study demonstrated that alternative splicing is the mechanism by which KAI1 undergoes downregulation in advanced cases of breast cancer because truncated/spliced KAI1 was correlated with more advanced cases of breast cancer. Another variant of KAI1 has been described as a mechanism for downregulation of this gene in advanced cases of breast cancer, and this involves insertion of intronic region of 274 bp between exons 8 and 9 (Mooez et al., 2011). Using video image processing to assess motility of cells in MDA-MB-231 breast cancer cell line, Miller et al. (2018) observed higher cell motion, as well as increased cell growth and proliferation, in cells that have the spliced variant of KAI1 compared to those expressing the wild type of KAI1 (Miller et al., 2018). Another reported mechanism that has been recently published is the role of one of the GTPases, known as RhoC GTPase, in reducing the gene expression of KAI1 in an invasive form of breast cancer (Xu et al., 2017). This study reported that two breast cancer cell lines were transfected with a specific anti-RhoC GTPase, following this, the authors observed an increase in the expression of KAI1. Interestingly, a study reported a relation between downregulation of KAI1 and expression of estrogen receptor in breast cancer tissue (Christgen et al., 2008). They postulated a role for estrogen receptor in repression of the KAI1 gene. The gene expression analysis with and without CD82 in microarray technique reveals the key role of KA11 in regulating prostate cancer and could be an effective biomarker for treatment and diagnosis purposes (Dodla et al., 2020).

## 6 Conclusion

The KAI1 gene is located on chromosome 11p11.2, and its protein product is a cell membrane glycoprotein that belongs to the transmembrane 4 superfamily. It is widely addressed as a metastasis suppressor gene in many cancer types, including breast cancer. Recent studies revealed altered expression levels of KAI1 in advanced cases of cancer suggesting that KAI1 might be a vital protein in the regulation of cancer metastasis and behavior. The low expression of the KAI1 might indicate a more aggressive form of breast cancer. Loss of KAI1 is taken as an important prognostic marker in forecasting the prognosis of breast cancer. However, only a limited number of studies about the role of KA11 in cancer patients is available. A better understanding of KAI1 function at the molecular level would help in developing prognostic biomarkers and therapeutics for breast cancer. Conclusively, this review has shed some light on the role, the putative mechanisms, and the level of expression of KAI1 in different types of cancer, particularly in breast cancer.

## Compliance with ethical standards: Research involving human participants and/or animals

This article does not contain any studies with human participants or animals performed by any of the authors.

## Funding statement

None.

## Declaration of Competing Interest

The authors declare that they have no known competing financial interests or personal relationships that could have appeared to influence the work reported in this paper.
